# Inequality in pandemic effects on school track placement and the role of social and academic embeddedness

**DOI:** 10.1038/s41539-024-00283-1

**Published:** 2024-11-27

**Authors:** Herman G. van de Werfhorst, Dieuwke Zwier, Sara Geven, Thijs Bol, Carla Haelermans

**Affiliations:** 1 European University Institute, Florence, Italy; 2 University of Amsterdam, Amsterdam, the Netherlands; 3 Maastricht University, the Netherlands, Netherlands Initiative for Education Research (NRO), and NOLAI, Radboud University, Nijmegen, the Netherlands

**Keywords:** Sociology, Education

## Abstract

Using register data and linked student-level sociometric survey data from the Netherlands, this study examines whether the impact of the COVID-19 pandemic on schooling outcomes (track recommendation and track enrollment in the seventh and ninth grades) is conditional on students’ academic and social embeddedness in the school setting. We estimated the counterfactual outcomes for the cohort that went through the school transition during the pandemic based on the outcomes of the pre-pandemic cohort, with similar earlier achievements, schools, and social backgrounds. Results show that the pandemic’s effect on tracking outcomes is weaker than its effect on student test scores elsewhere reported. Nevertheless, the pandemic has had stronger adverse impact on disadvantaged students. Moreover, student self-efficacy, academic motivation, and parental involvement are related to more negligible negative pandemic effects on schooling outcomes. We find no evidence for an association between student grit or parental network centrality and the magnitude of estimated pandemic effects.

## Introduction

The COVID-19 pandemic has had detrimental effects on learning progression, especially for students from disadvantaged backgrounds^1–4^. Inequalities in educational outcomes by socioeconomic background have grown. Furthermore, learning delays have been estimated to negatively affect life expectancy and economic growth^5,6^, implying long-term human capital and well-being losses. A thorough study of factors contributing to learning losses is crucial for schools, school boards, governments, and scientists to help target possible restoration efforts to reduce learning delays and to cope with potential new school closures.

While several studies have investigated the impact of the pandemic on schooling outcomes (for a meta-analysis, see ref. ^7^), there are two blind spots in the literature. First, most studies focus on student test scores, while the impact of the pandemic on critical school transitions has yet to be studied. From a life course perspective, final schooling attainment unfolding along educational transitions is arguably a more important outcome than test scores.

Second, more information about possible explanations for such school closure-related gaps is needed. A promising set of explanations refers to students’ social and academic embeddedness, and a recent paper has called for research on this^8^. With embeddedness, we refer to the sociological understanding that social integration in intermediary organizations and networks fosters compliance with dominant norms, provides information, and enables the sharing of resources. Such embeddedness can, first, be of a *social* kind: if parents are well-integrated in parental networks in school, the available social capital is held to avoid students’ deviant behavior, promote student learning, and advance the spread of information on school matters^9,10^. Second, embeddedness can be *academic*; children who are highly academically motivated, have higher levels of self-efficacy, and show grit, have embodied the pro-school orientations that help their learning gains^11–14^. Such psychological traits may mitigate the potential damage the COVID-19 pandemic can bring to school careers.

Social and academic embeddedness, like other types of resources, are likely to be especially relevant for learning when schools were closed for in-person education, and moved their instruction to online environments^8,15^. Students had to rely more on themselves and their parents, and adopting pro-school norms through social and academic integration likely helped cope with the disrupted educational process. Additionally, well-connected parents can more easily organize access to online school resources or rely on other parents to help with home-schooling. Thus, while recent (quasi-)experimental research has shown weak effects of intergenerational social capital on student learning in regular settings^16,17^, the resources and norms distilled from such networks may be particularly relevant in the context of disrupted learning in pandemic times.

**Fig. 1:**
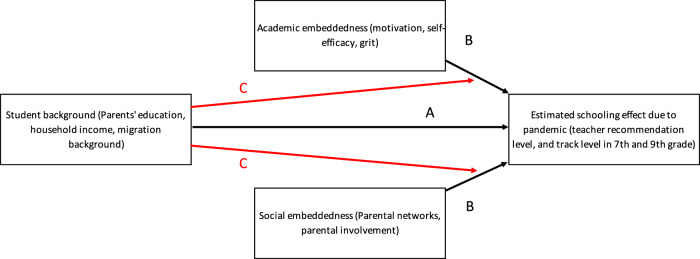
The theoretical model. This figure shows the main interests of the paper, which is to calculate the pandemic effect on tracking outcomes by socioeconomic background (**A**), the association between embeddedness and the size of the pandemic effect (**B**), and the moderation of the embeddedness parameters by socioeconomic background (**C**).

More specifically, our study objectives are threefold. First, we assess to what extent the pandemic has affected the transition from primary to secondary school for students of different socioeconomic backgrounds (arrow A in Fig. 1). We study this transition in the Netherlands, where students enter the tracked secondary system after grade 6. Second, we examine whether various types of embeddedness are associated with how much the COVID-19 pandemic has affected schooling outcomes (arrows B in Fig. 1). For instance, students more strongly motivated to learn may have suffered less, or children of parents who are well-connected to other parents may have found ways to cope with the challenges of out-of-school learning.

Third, we explore potential heterogeneity in the relationship between embeddedness and pandemic effects by socioeconomic background (arrows C in Fig. 1). Such a pattern could be found if embeddedness forms a buffer against schooling delays, especially for children of disadvantaged backgrounds. Or, reversely, if children from advantaged backgrounds benefit disproportionately from social and academic embeddedness, inequalities in pandemic effects magnify through differences in embeddedness. In analytical terms, we examine whether the association between embeddedness and pandemic impact on schooling outcomes is moderated by socioeconomic background.

Using a unique combination of survey and register-based datasets from the Netherlands, we study the association between social and academic embeddedness and learning delays due to the pandemic. The Netherlands forms an interesting case to research tracking outcomes: it has an early-tracking education system where students are placed in multi-year tracks at 12, preparing for their qualification level. Moreover, the Dutch system uses standardized tests over multiple primary school years and has a standardized end-of-school test to inform the transition to secondary school. The end-of-school test was canceled during the first wave of the pandemic, so there was a sudden ‘destandardization’ of the transition from primary to secondary school that may have affected students of different socioeconomic backgrounds differently.

We follow a recent scholarship that estimates the impact of the pandemic on schooling outcomes by comparing the affected cohort with previous cohorts^1–3,18–21^. Our main contributions are twofold. First, we move beyond the study of test scores and focus on a crucial educational transition from primary to a tracked secondary education system. More specifically, we study the teacher recommendation for tracks, and factual placement in different tracks in secondary education. Second, unlike previous studies, we explore potential explanations for differences in students’ learning losses by associating the estimated pandemic effects on schooling outcomes with information on students’ social and academic embeddedness. This part of the analysis is descriptive. Since survey data on embeddedness is unavailable for the pre-pandemic cohort and thus not included in the counterfactual analysis, causal statements cannot be made. Nonetheless, these survey data, collected right before the pandemic outbreak, offer a better opportunity to explore factors that may contribute to or mitigate students’ learning delays compared to prior research that relied exclusively on administrative data and thus lacked attitudinal and sociometric measures. This way, our study contributes to understanding the mechanisms explaining pandemic effects on education, of which little knowledge exists.

## Results

### Describing the pandemic effect

We study the pandemic effect on three schooling outcomes related to the transition from primary to secondary education in the tracked Dutch education system: the track that is recommended to students by the primary school teacher, the track level in which students eventually enroll the following year (seventh grade), and the track they have enrolled in the third year of secondary education (ninth grade). In the Dutch system, the transition from primary to the tracked secondary system is based on a combination of teacher recommendation and a standardized end-of-school test. A first preliminary track recommendation is given before the test, and the final recommendation can (but not necessarily will) be adjusted upwards if the end-of-school test indicates a higher recommendation (downward adjustments are not allowed). The standardized end-of-school test was canceled in the first wave of the pandemic, so the preliminary recommendation (given early March 2020) automatically became the final one. If the pandemic affected the track recommendation, this happened through the absence of adjustment.

Research shows that, in pre-pandemic times, there was a systematic relationship between student socioeconomic background and the deviation between the track recommendation and the student test score: advantaged students get a higher recommendation than would result from the end-of-school test, and disadvantaged students get a lower recommendation^22^. The cancellation of the test has, thus, likely affected disadvantaged students disproportionally, as they saw their opportunity for adjustment of their preliminary recommendation disappear. Two outcomes have fine-grained scales, including combinations of tracks, ranging from 0–8 (track recommendation) and 0–7 (track enrollment; see Methods for details), and the third distinguishes the three main tracks existing in ninth grade.

To assess the pandemic effect, we use a counterfactual approach that compares the factual outcome with the counterfactual outcome had there not been a pandemic. To estimate this counterfactual outcome, we use students of the cohort one year before the outbreak of the pandemic, with identical test scores in the two preceding grades, in schools with equal levels of academic performance, of the same socioeconomic background, and of the same gender. Due to the availability of test score data in the national register data, we can use about 27 percent of each cohort (i.e. around 50,000 students per cohort, see Methods for details).

Table 1 shows the estimated pandemic effects, averaged for all students and by socioeconomic background. Socioeconomic background is measured by a combination of parental education and household income to reflect different types of resources in the family. Both can be comparatively high and comparatively low, education can be high but income relatively low, and income can be comparatively high and education low. A negative score indicates that the schooling outcome was lower for the pandemic cohort than in the counterfactual in the absence of a pandemic, and a positive score means that the outcome was higher than in the counterfactual. The average pandemic effect is -0.068 levels for track recommendation, -0.039 levels for seventh-grade track enrollment, and +0.011 levels for ninth-grade track level. These are small but statistically significant effects. Suppose we scale these effects in terms of standard deviations of the ranked outcome variables to make comparisons possible to published effects on test scores. In that case, these effects equal -0.028, -0.019, and +0.013 standard deviations, respectively (Table 1).

**Table 1:** Estimated pandemic effects on three educational outcomes

		Averaged over all students		Lower income and no parent with a college degree		Medium/high income, no parent with a college degree		Lower income, at least one parent with a college degree		Higher income, at least one parent with a college degree	
Track recommendation level	estimated pandemic effect	−0.068	***	−0.111	***	−0.072	***	−0.058	***	−0.031	***
	bootstrapped s.e.	0.005		0.011		0.013		0.014		0.009	
	effect size in s.d.	−0.028		−0.046		−0.030		−0.024		−0.013	
Secondary school track 7th grade	estimated pandemic effect	−0.039	***	−0.083	***	−0.050	***	−0.008		−0.011	
	bootstrapped s.e.	0.006		0.014		0.013		0.015		0.009	
	effect size in s.d.	−0.019		−0.041		−0.024		−0.004		−0.006	
Secondary school track 9th grade	estimated pandemic effect	0.011	***	−0.001		0.014	**	0.015	*	0.015	**
	bootstrapped s.e.	0.003		0.006		0.005		0.008		0.006	
	effect size in s.d.	0.013		−0.001		0.018		0.019		0.019	
N (2018)		46,893		14,335		10,011		9492		13,055	
N (2019)		50,313		15,123		10,620		10,078		14,492	

***p < 0.001.

**p < 0.01.

*p < 0.05.

bootstrapped 50 times.

The overall pandemic effect on tracking outcomes is thus smaller than the effects on test scores, as reported in a meta-analysis (-0.14)^7^. This implies that known learning delays did not automatically translate into lower-track placements. In fact, the pandemic has a slight positive effect on students’ ninth-grade track enrollment. Interestingly, if we look at Fig. 6, the average primary school performance of students in the pre-university track went down between the pre-pandemic and the pandemic cohort. This explains the positive causal effect of the pandemic as we compare students with equal performance levels. One possible reason for this could be that secondary schools worked within their existing tracking structure and found, encouraged by the ministry to do so, ways to correct the mild negative effect at the start of secondary school by lowering the bar for getting the higher option. Also, the Dutch government has invested a lot of resources into the recovery of student performance after the pandemic, which may have raised student performance, avoiding downstreaming in lower secondary education. We discuss these interpretations at more length in the Discussion.

The negative pandemic effect on the first two schooling outcomes is the largest for students from socioeconomically disadvantaged backgrounds (-0.111 and -0.083 levels, respectively). This group has no effect on the ninth-grade track level, while the effect is around +0.015 levels for the three other SES groups. There is no discernable pandemic effect on track enrollment for students with parent(s) with a college degree.

**Fig. 2:**
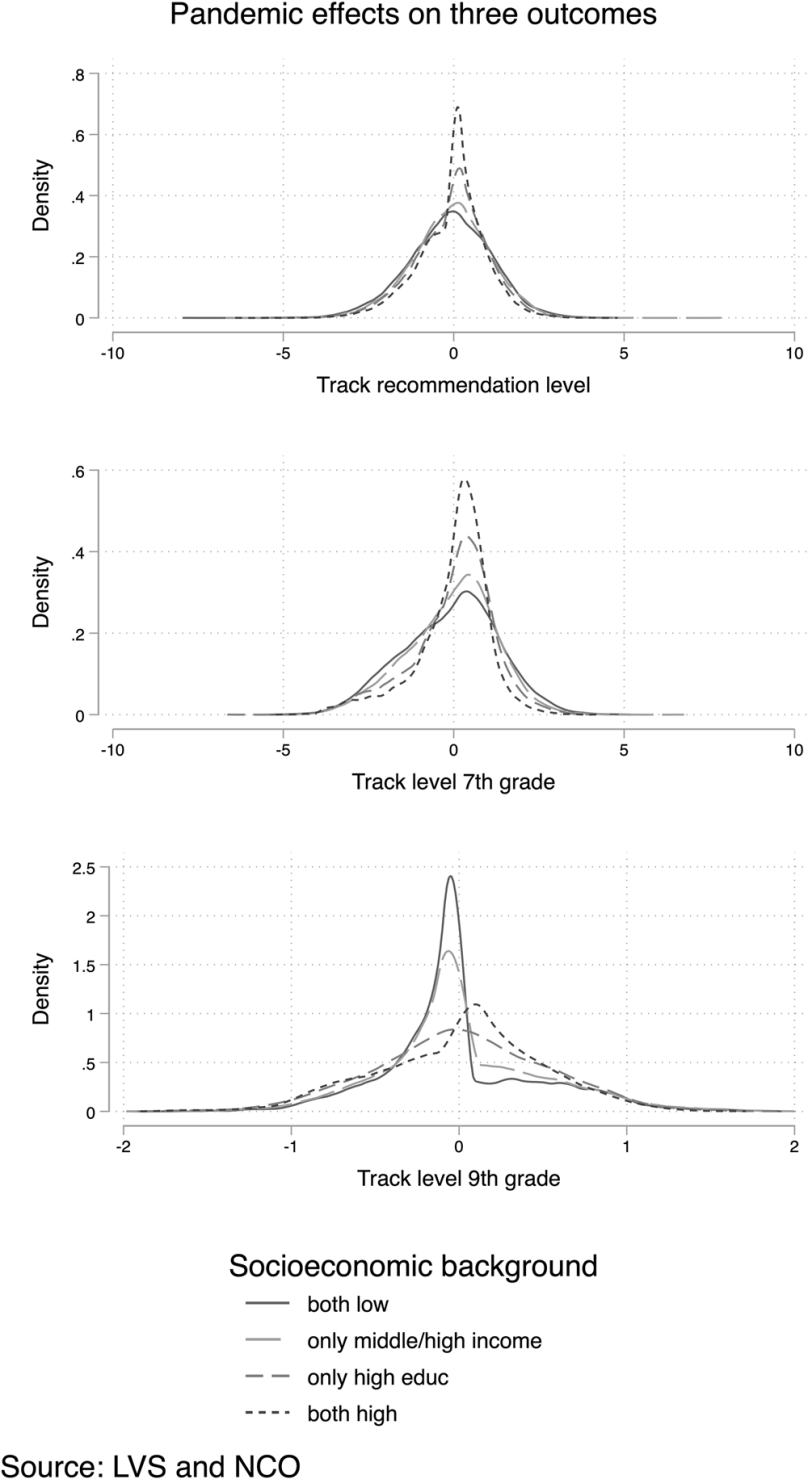
The size of the pandemic effects on three educational outcomes by socioeconomic background. This figure shows the individual estimated pandemic effect by socioeconomic background, based on the comparison of the factual outcome of the cohort that went through the transition to secondary school in the pandemic and the counterfactual outcome based on the pre-pandemic cohort with similar levels of achievement, gender, schools, and socioeconomic backgrounds.

Figure 2 shows kernel density plots of these pandemic effects on the three schooling outcomes by socioeconomic background. The pandemic impact is not only minor for students from more advantaged backgrounds but also has a smaller dispersion. A more considerable variability of pandemic effects is found for children of less advantaged families regarding track recommendation and seventh-grade track level. Thus, children from a socioeconomic background with a lower income and no parent with a college degree have a larger dispersion, especially at the lower end of the distribution (i.e., with negative pandemic effects; the solid line lies above the others in the negative range of the pandemic effect). With regard to ninth-grade track level, the least advantaged children experienced a positive pandemic effect less frequently than the other groups. Conversely, children of the most advantaged group, with a higher income and parent(s) with a college degree, experience positive pandemic effects more frequently and consistently across the distribution.

### Embeddedness and the pandemic effect on schooling outcomes

Our second main interest is the relationship between social and academic embeddedness and the magnitude of the pandemic effects. For this, we can use the matched data set (*N* = 402) that combines the register data used to assess the pandemic effects as described earlier and the student survey data collected right before the pandemic outbreak.

**Fig. 3:**
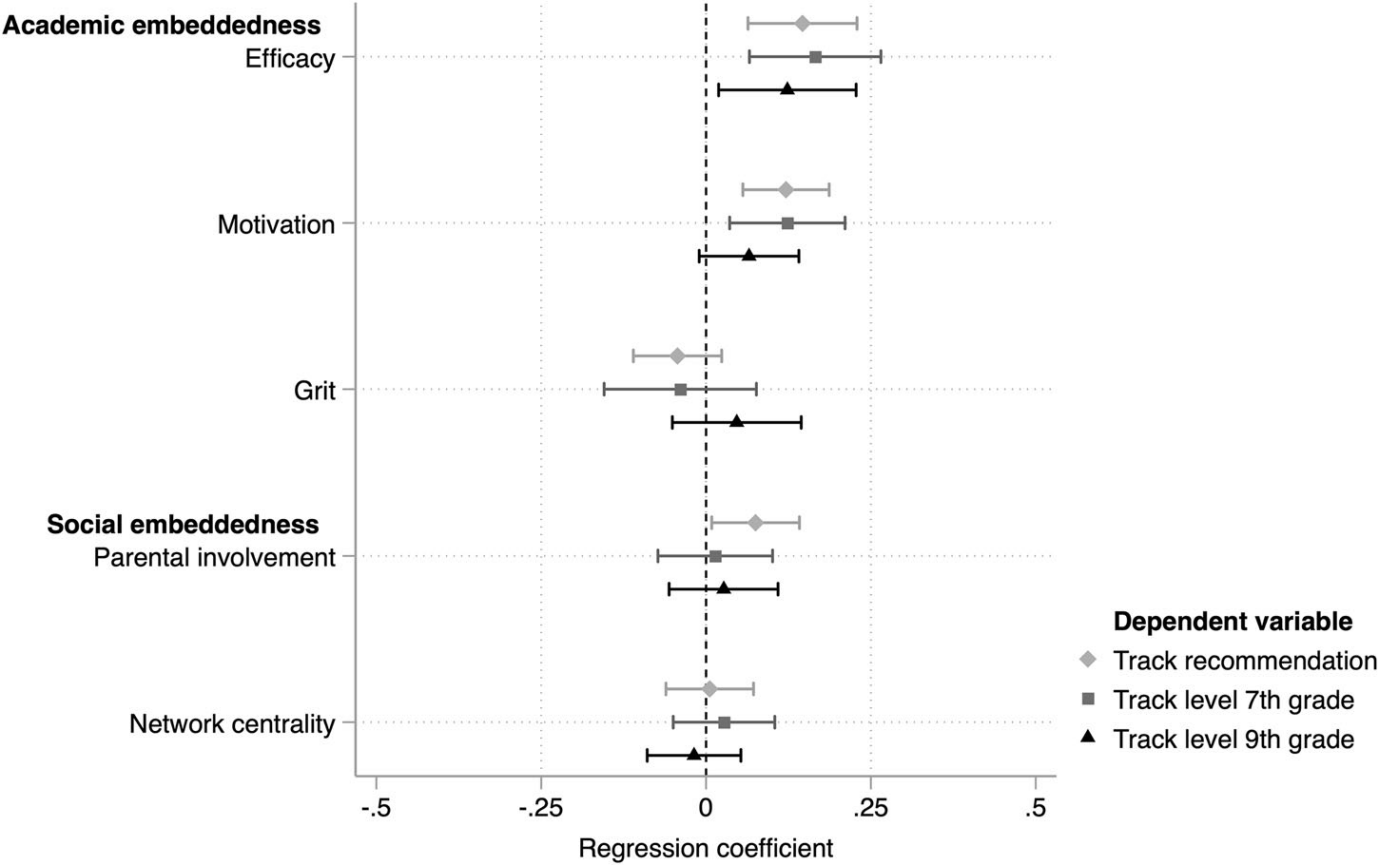
Relationship between academic and social embeddedness and the size of the pandemic effect. This figure shows the regression coefficients of embeddedness variables predicting the three outcomes, holding constant on the counterfactual outcome based on the pre-pandemic cohort with similar achievement levels, gender, schools, and socioeconomic backgrounds.

Figure 3 shows the coefficients from structural equation models of the five embeddedness indicators, all from separate models (precise coefficients in the Online Supplement Table 4). Student self-efficacy and academic motivation are associated with more minor learning delays due to the pandemic. The standardized regression coefficient of student self-efficacy on track recommendation is 0.15, implying that one standard deviation increase in self-efficacy is associated with 0.15 higher standard deviations in track recommendation, holding constant the counterfactual outcome had there been no pandemic. For academic motivation, the association equals 0.12 standard deviations; for parental involvement, there is also a statistically significant estimate of 0.08 standard deviations. It should be emphasized that, since counterfactual outcomes are not adjusted for student embeddedness levels, these results are to be interpreted descriptively. Student grit and parental network centrality are unrelated to estimated pandemic effects. Regarding secondary school track in seventh grade, similar patterns emerge for efficacy and motivation, although parental involvement is not associated with the size of the pandemic effect. Concerning ninth-grade track level, only student self-efficacy shows a statistically significant association, with a coefficient of 0.12.

### Embeddedness and pandemic effects by socioeconomic background

**Fig. 4:**
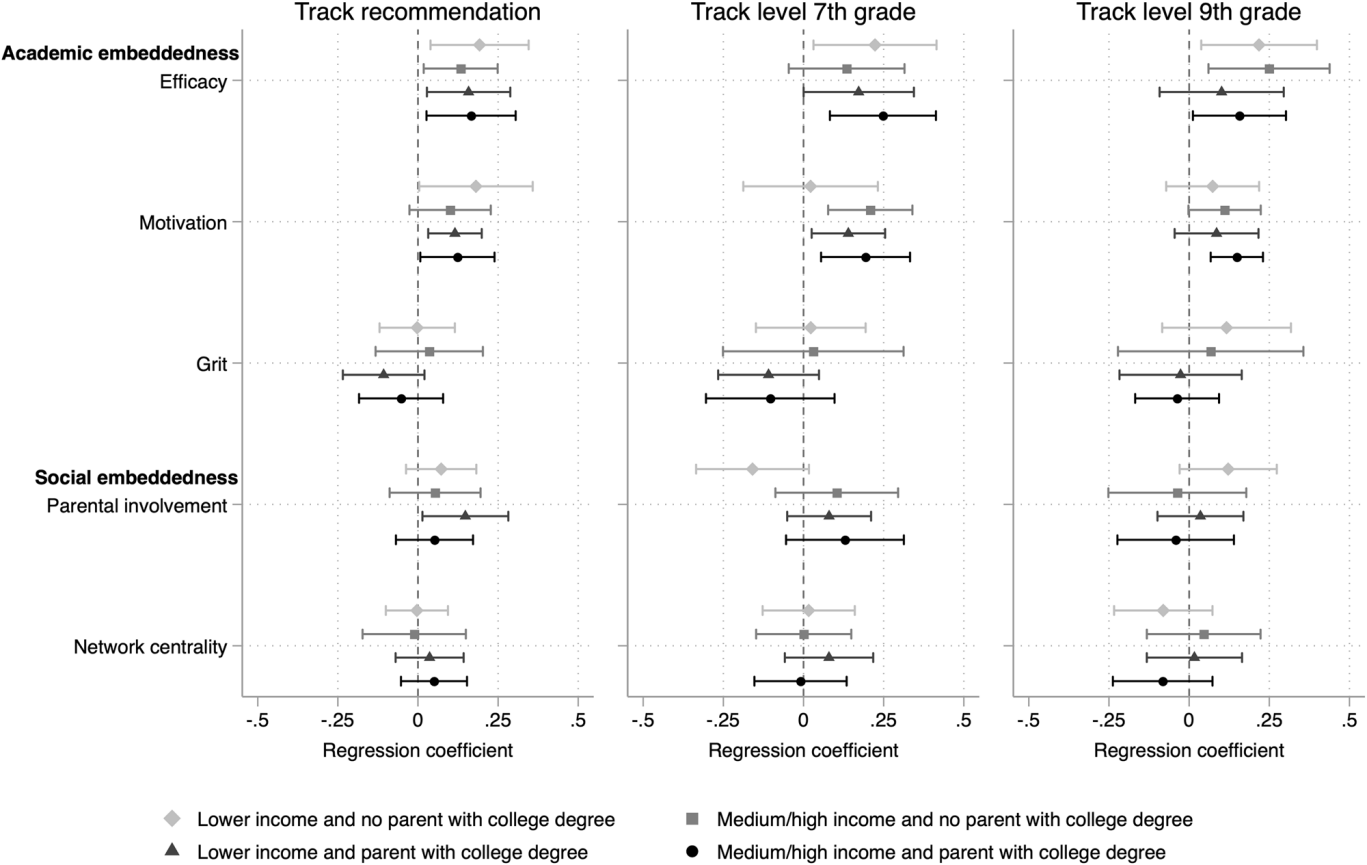
Heterogeneity in the relationship between academic and social embeddedness and pandemic effects by socioeconomic background. This figure shows the regression coefficients predicting the three outcomes, holding constant for the counterfactual outcome based on the pre-pandemic cohort with similar achievement levels, gender, schools, and socioeconomic backgrounds, separately by socioeconomic background.

The third study objective is to examine whether embeddedness matters differently for students from different socioeconomic backgrounds. Figure 4 shows the multigroup structural equation model (precise coefficients in Supplementary Table 4). The multigroup model fitted the data better than the single-group model, suggesting that exploring heterogeneity in the embeddedness correlates of pandemic effects by socioeconomic background is valuable. However, despite the fit statistics favoring the model separating effects by socioeconomic background, Fig. 4 shows that point estimates for most embeddedness indicators are pretty consistent across the socioeconomic groups, with overlapping confidence intervals. In other words, we do not find strong evidence of heterogeneity based on socioeconomic background in the association between academic and social embeddedness and pandemic effects.

While the overall picture reveals limited heterogeneity based on socioeconomic background, a few results are worth discussing. Self-efficacy is associated with more minor pandemic effects across all three schooling outcomes and most socioeconomic groups. This is also true for motivation, except for the near-zero coefficient among the most disadvantaged students on the seventh-grade track level, which is positive for the other socioeconomic groups. Group-specific estimates for parental involvement on this schooling outcome show a similar pattern, though none of the coefficients is statistically significant. Generally, there is greater socioeconomic heterogeneity in parental involvement estimates on track level compared to track recommendation. As in earlier results, student grit and parental network centrality are not significantly associated with pandemic effects for any socioeconomic group or schooling outcome.

## Discussion

A sizeable body of research has emerged on the effects of school closures or other pandemic-related factors on student achievement. While such research is relevant, we addressed two gaps in the current literature. A first shortcoming of the current literature is that pandemic effects have mainly been studied concerning academic achievements, with little attention given to educational transitions. The level of educational attainment is arguably a more relevant factor than academic achievement in the intergenerational reproduction of advantage, and social origin effects are larger on attainment^23^. Given that educational attainment unfolds across several transitions in the school career, which can be seen as branching points where continuation decisions are made, studying the impact of the pandemic on educational transitions is highly relevant to understand further how external shocks may impact societal inequalities. In contrast to previous studies, we focus on the transition from primary to secondary education and investigate whether making that transition during the first wave of the pandemic resulted in larger inequalities than in pre-pandemic times. We do this for the Netherlands, which has an early-tracking educational system where students enroll in a prevocational, general, or pre-university track after sixth grade.

In this study, we used a unique combination of student-level sociometric survey data and register data from the Netherlands to examine the effects of the pandemic on tracking outcomes in secondary school and whether these pandemic effects are conditional on students’ academic and social embeddedness in the school setting. We estimated the impact of the pandemic on track recommendation and track enrollment with a counterfactual approach based on a comparison of cohorts. We associated the estimated pandemic effects with survey information obtained right before the outbreak of the pandemic. Addressing our first study objective of assessing the pandemic effect on tracking outcomes, the results showed that the pandemic has especially hit disadvantaged students. However, the overall effect on tracking outcomes was not very strong. The pandemic effect is weaker than on student test scores as published elsewhere, at least in terms of standard deviations in the outcome variable^2,7^. Notably, the pandemic effect was slightly positive on the ninth-grade track placement, except for students from low-educated, below-average income backgrounds (for whom the effect was zero). It appears that mainly the pre-university track has lowered its standards in terms of (pre-pandemic) primary school performance.

One should interpret these results within the specific context of the Dutch educational system. Pandemic effects on tracking outcomes may be minor compared to those on test scores because students had to fit in the existing structure of the educational system, with usually high stability of the proportions of students allocated to different tracks. Even if a shock like the pandemic emerges, schools work within this system, plausibly aiming for little distortion of the proportions of students going into the different tracks. It should be noted that primary schools were actively encouraged by the Ministry of Education to give more generous recommendations in the pandemic cohort. The ninth-grade effect was slightly positive, which resulted from students entering the pre-university track with lower average primary school performance than before, which fits with this interpretation. Further research could more explicitly study secondary school-level factors that contributed to the size of the pandemic effects during the progression through the grades.

Another important contextual factor that should be kept in mind is that there has been an active policy to reduce the impact of the pandemic on schooling outcomes, with a website built by the Ministry of Education listing evidence-based measures that schools could take, and a large budget spent on reparation efforts (a one-time reservation of EUR 8.5 billion, on a yearly ministerial budget of around 41 billion for Education, Science and Culture as a whole). These efforts may have successfully diminished the pandemic effect on students in different tracks. Given that schools vary significantly in how well they are prepared to educate students effectively during the pandemic^24,25^, it may be the academic schools that have been most effective in using the additional resources.

Lastly, the positive pandemic effects on ninth-grade track placement may be caused by the fact that the control group, who went through the transition pre-pandemic, did experience the effects of the pandemic once they were in ninth grade. They were in the first year of secondary school when the pandemic broke out, which may have led to disadvantages that the “treatment cohort” did not experience.

Our results show that the pandemic has hit students differently, depending on their parental educational level and household income. While the average adverse effects of the pandemic were modest, we found that the effects were more strongly negative for students from the least advantaged families. Thus, while the effects were weaker than published results on academic achievement, we do find that the pandemic has enlarged existing inequalities in the transition from primary to secondary education. The long-term effects of such minor effects on track enrollment can be large, given that track placement is a strong predictor of graduating from higher education, which, in turn, leads to large returns on the labor market.

A second shortcoming in the literature that we addressed is that more needs to be known about possible explanations for pandemic effects on student outcomes. The broader literature shows that academic motivation, self-efficacy, and grit positively affect school performance. We conceptualized these as indicators of academic embeddedness, as they represent critical psychological traits linked to a positive school orientation. Moreover, students may also benefit from social embeddedness, i.e., having parents who are involved in the schooling process of their children and are well-connected to other parents.

Our second research objective was to associate the size of the pandemic effects with embeddedness. Our results showed that the impact of the pandemic on the transition process from primary to secondary school is correlated with academic and (to a lesser extent) social embeddedness. Student self-efficacy and motivation are associated with more minor negative effects due to the pandemic. It is known that teachers base their recommendations on traits such as student motivation and self-efficacy^26^. The destandardization that happened in the pandemic year, with the cancellation of the standardized test known for its equalizing effect, may have increased the importance of teachers’ evaluations of such student traits.

Regarding social embeddedness, parental involvement is correlated with the size of the pandemic effect on track recommendation. At the same time, no evidence is found for a relation between parents’ integration in school networks and pandemic effects. Furthermore, we find limited evidence of heterogeneity in the relationship between social and academic embeddedness and pandemic effects by socioeconomic background, a heterogeneity that was our third research objective. This suggests that the potential buffering effect of these factors against pandemic-related learning delays is not limited to a specific subgroup of students. Academic embeddedness is associated with more minor pandemic effects, and social embeddedness seemed less relevant. Future research may further conceptualize and examine these two forms of embeddedness.

From a sociological perspective, it is noteworthy that we find no evidence of a relationship between parental network integration and the extent to which children were affected by the pandemic. Recent scholarship has questioned the causal effects of intergenerational closure, dense networks of parents around the school enabling the enforcement of pro-school norms^16,17^. Our null findings for parental network integration align with those studies and suggest that social embeddedness in parental networks may be less relevant than has been theorized in the social capital literature^9,27^. Future research could further critically engage with social capital effects in school careers.

Although our study contributes to the literature by being one of the first to address the effects on school transitions and to explore possible explanations for differences in pandemic effects among students, some limitations should be acknowledged. First, as our design exploits the overlap between a survey and standardized student assessment data, we used a sample of *N* = 402 for part of the analysis. This number of observations is sufficient to detect significant student-level predictors of the size of the pandemic impact. Still, the relatively small sample size is a limitation of our study. Due to this limitation, we were, for example, unable to perform further heterogeneity analyses. For future research, it is recommended to have a sufficient sample size to further dive into the heterogeneity effects of the social and academic embeddedness factors.

Furthermore, the second part of our analysis precludes causal statements. Since embeddedness data cannot be included in the counterfactual analysis, we cannot be sure whether the identified embeddedness correlates form buffers against pandemic effects, as theorized, or if observed associations (partially) reflect confounding factors. This can be problematic as student motivation is associated with later achievements, even controlled for earlier achievement^28^. Nevertheless, our analysis of the causal effect of the pandemic is within-group (by SES, gender, and school-level performance), so such group characteristics may capture a sizeable part of this conditional association between motivation and later performance. Hence, since related research lacks pre-pandemic attitudinal and sociometric measures, this descriptive endeavor still brings us closer to understanding the mechanisms behind pandemic effects in education, of which little knowledge exists.

In summary, the effects of the pandemic were not very strong in tracking outcomes, but small differences in socioeconomic background could translate into further inequalities in school careers. There is a known causal effect of track placement on learning outcomes^29^, and given that early-tracking education systems like in the Netherlands typically create path dependencies in further learning opportunities^30^, the small socioeconomic gaps in the pandemic effect on the transition to secondary school can turn into a significant impact on future life courses. Furthermore, given that student and family factors are related to the size of the pandemic effects, our study demonstrated that there is more heterogeneity in pandemic effects than other studies have been able to show. Furthermore, our results suggest that the impact of the pandemic may be weaker for students who display higher levels of motivation and self-efficacy and have parents who are intensely involved with their schooling.

## Methods

### Data

**Fig. 5:**
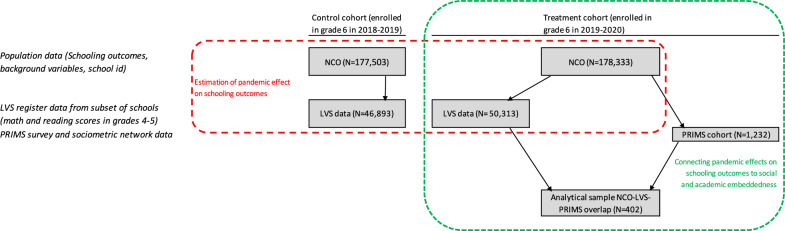
Overview of datasets and their connection. This figure shows how the various datasets are connected and which sample sizes remain after integrating them. The register datasets within the dotted line in red are used to estimate the pandemic effect, and the datasets within the dotted line in green are used to assess the association between student embeddedness and the estimated pandemic effect.

We merge three different datasets at the individual (student) level. The results are based on calculations by the authors using non-public microdata from Statistics Netherlands. See Fig. 5 for an overview of how the datasets are connected.

First, we use register data for all students in Dutch primary schools who were in their final year (sixth grade; we use internationally recognized grade numbers throughout this paper, although the Dutch grade numbers are different). We use data from (internationally used) grades 4-6 in primary schools, and seventh and ninth grade in secondary schools. These data are available in the Netherlands Cohort Study on Education (NCO)^31^. The cohort in sixth grade in the school year 2019-2020 is the treatment cohort, whose learning outcomes may have been affected by the school closures due to the COVID-19 pandemic. The previous cohort (sixth grade in 2018-2019) forms the control group, on which we estimate the expected schooling outcomes for the treatment cohort had there not been a pandemic. From the NCO data, we retrieve student background information (parents’ education, household income, and gender), as well as the dependent variables (track recommendation and track enrollment).

The second dataset we use is the longitudinal standardized student monitoring system (LVS, *Leerling Volg Systeem* in Dutch), which includes standardized tests for students in grades 4 and 5. We use the mid-year tests of grades 4 and 5 taken around February (there are also end-of-year tests, but these had a lot of missing values on reading comprehension). We can use the LVS data of the largest provider of standardized tests, for a selection of schools that took part in a national COVID study on behalf of the Netherlands Initiative for Education Research, part of the leading national research funding agency Netherlands Organisation for Scientific Research^2^. The pilot covers 26 (control cohort) and 28 (treatment cohort) percent of all students in the Netherlands. While primary schools are obliged to use a student monitoring system, there are multiple private sector suppliers for this service. The largest is Cito, and only the Cito student monitoring system has been made available for research in the register data environment. This has to do with the limited comparability of the tests of various suppliers. We used mathematics and reading comprehension tests in grades 4-5 of the treatment and control cohort.

The third dataset is a top-up survey of the NCO, called PRIMS (the transition from PRIMary to Secondary education), of which the first wave was coincidentally fielded right before the first school closure (data collected in January/February 2020)^32^. The PRIMS data are collected among 1,232 sixth graders in a representative sample of 66 Dutch primary schools. The PRIMS data include student survey data and sociometric student and parental contact network measures. The combination of register data and the PRIMS survey allows us to study in more detail how estimated pandemic effects are conditional on students’ academic and social embeddedness.

The collection and use of PRIMS data for the current study have received ethical approval from the University of Amsterdam, project number 2019-AISSR-10381. This includes informed consent given by the parents or legal guardian. Use of administrative data of NCO and LVS was approved by Statistics Netherlands for project 9232 “Sociale ongelijkheid en veranderingen in levenslopen.”

The Online Supplement Table 1 shows t-tests of group differences between the used datasets on key variables. We use entropy balancing to weigh the PRIMS analytical sample to the LVS/NCO register data of the same cohort concerning the averages on grades 4 and 5 mathematics and reading scores, household income, parents’ education, gender, and migration status^33,34^. The resulting weight variable has a mean of 1 and a standard deviation of 0.45, which are used in the structural equation models.

Altogether, the sample size of *N* = 402 gives an accurate picture of Dutch students. We focus primarily on effect sizes and 95 percent confidence intervals and follow good practice of not focusing too rigidly on significance levels.

### The Model

#### Assessing the impact of the pandemic

We calculate, for the treatment cohort, the expected (counterfactual) learning outcomes if the pandemic had not happened based on the schooling outcomes of similar students in the same schools of the control group. We identify identical students based on the student’s earlier performance on standardized tests, in the same grades, and schools of the same average academic performance in math and reading.

To achieve this, the first step (Eq. 1) is to estimate the statistical relationship between student schooling outcome *Y* of student *i* in school *j*, for control cohort *k0* (i.e., the cohort preceding the treatment cohort), student previous performance *P* in grades 4-5 (on both math and reading, so four performance indicators), and student (binary) gender *G*. We employ hybrid models, meaning that for all predictor variables we included school means (between component) and individual deviation from the school mean (within component) (Allison 2009). Moreover, we included interaction terms between student and school-level performances, allowing for heterogeneous learning outcomes across schools for students at different performance levels. The predicted score of the pre-pandemic cohort on the dependent variable can be seen as the counterfactual outcome, to which we will later compare the factual outcome of the treatment cohort that went through the school transition during the pandemic. To assess this counterfactual performance, the model implies that we compare students within each socioeconomic group separately based on their performance, their school’s average performance, and their interaction, and the student’s gender, the gender composition of the school, and their interaction. An alternative specification would be to include school fixed effects, but school fixed effects ordinal logit models cannot be estimated on the large register data. Furthermore, there are problems with fixed effects non-linear models^35^.

Given the ordinal measurement level of the dependent variable, we used an ordinal logit model for this equation. For each level *m* in the ordinal scale, the log odds to score that level or higher, relative to lower, is estimated as a function of regressors, with assumed identical gamma coefficients across the estimated steps. Note that these models are estimated separately by socioeconomic background *c*, as we can later compare the counterfactual outcome with ${E(\varepsilon }_{{ij}.c})=0$ with the background-specific factual outcome during the pandemic. The predicted track recommendation or enrollment level is calculated in the following way. The ordinal logit model yields predicted probabilities of each of the categories of the dependent variables, depending on individual and school-level covariates. To arrive at one predicted outcome, we multiplied each category’s probability with the outcome variable’s rank order value. This way, we quantified the ordinal variable with a range from 0–8 (track recommendation), 0-7 (seventh-grade track enrollment), and 0–2 (ninth-grade track enrollment). With the current approach, we obtain a predicted (i.e., counterfactual) outcome with the same range as the observed outcome. In contrast, a linear model could result in predicted levels outside of the empirical range of the data.

1 $$\begin{array}{l}{\mathrm{ln}\left(\frac{p(y\ge m)}{p(y < m)}\right)}_{{ij}}^{k0.c}={\alpha }_{m.{ij}}+{\gamma }_{1}{P}_{j}^{{between}}+{\gamma }_{2}{P}_{{ij}}^{{within}}+{\gamma }_{1}{P}_{j}^{{between}}* {P}_{{ij}}^{{within}}\\\qquad\qquad\qquad\qquad\quad\;\;\;+\,{\gamma }_{3}{G}_{j}^{{between}}+{\gamma }_{3}{G}_{{ij}}^{{within}}+{\varepsilon }_{{ij}.c}\end{array}$$

Then, in a second step (Eq. 2), we calculate the counterfactual student learning outcome for the treatment cohort $\widehat{{Y}_{{ij}}^{k1.c}}$ (i.e., the cohort affected by the pandemic in their final year of primary school), based on model predictions based on the previous cohort for same-gender children in the same grade, with the same individual and school-level performance. Again, we do this separately by socioeconomic background *c* because the expected outcome will likely vary across socioeconomic backgrounds within the same school level and individual performance.

2 $$\left(\widehat{{Y}_{{ij}}^{k1.c}}|P,G,\,J\right)=\left(\widehat{{Y}_{{ij}}^{k0.\,c}}|P,G,\,J\right)$$

Then, in a third step (Eq. 3), we calculate the estimated effect of the pandemic ${\delta }_{{ij}.c}$ as the difference between the observed schooling outcome ${Y}_{{ij}}^{k1.c}$ and the counterfactual schooling outcome $\widehat{{Y}_{{ij}}^{k1.c}}$ had there not been a pandemic. This effect is measured in units of the track recommendation level or track enrollment. These ${\delta }_{{ij}.c}$ are reported in Fig. 2.

3 $${\delta }_{{ij}.c}={Y}_{{ij}}^{k1,\,c\,}-\widehat{{Y}_{{ij}}^{k1.c}}$$

#### Linking the pandemic effect to embeddedness

After establishing the pandemic’s impact on schooling outcomes, we turn to the analysis of the relationship with student embeddedness. Supplementary Figure 1 in the Supplementary file shows the distribution of the pandemic effects for the PRIMS subsample and the LVS sample described above. We model the association with embeddedness by predicting the schooling outcome$\,{Y}_{{ij}}^{k1}$ for treatment cohort k1 for individual *i* in school *j*, and including the counterfactual outcome $\widehat{{Y}_{{ij}}^{k1.c}}$ as predictor (Eq. 4). The main predictors of the models are the embeddedness variables *E* (student self-efficacy, student motivation, student grit, parental involvement, and parental network centrality), which we include in separate models). The models are estimated using structural equation models and employ clustered standard errors at the classroom level. The results of the models of Eq. (4) are displayed in Fig. 3. Because the effect of the pandemic is separately estimated by socioeconomic groups, mediation of a possible SES gap in pandemic effects by indicators of embeddedness is not something that can be studied in a meaningful way. It should be noted that student traits like motivation and work habits hardly mediated SES effects on teacher expectations in the Netherlands^36^.

4 $${Y}_{{ij}}^{k1}={\alpha }_{{ij}}+{\gamma }_{1}\widehat{{Y}_{{ij}}^{k1.c}}+{\gamma }_{3}{E}_{{ij}}+{\varepsilon }_{{ij}}$$

Finally, we also estimate the model of Eq. (4) as a multiple group model with separate estimates for the embeddedness variables by socioeconomic group. In the Online Supplement, we provide information on the Confirmatory Factor Analyses (CFAs) of our scales and tests of measurement invariance across socioeconomic groups (Supplementary Table 2). The Online Supplement also shows fit statistics comparing the multigroup and single-group model (Supplementary Table 3). These fit statistics generally support estimating heterogeneous embeddedness effects across socioeconomic groups. The heterogeneous effects are displayed in Fig. 4.

### Dependent variables

We study pandemic effects on three educational outcomes: the track recommendation by the primary school teacher, track enrollment in the first year of secondary school (seventh grade), and track enrollment in the third year of secondary school (ninth grade).

The track recommendation is an essential element in the allocation process from primary to secondary schools and is unequally distributed even after holding constant for test scores^37^. In the Dutch system, the preliminary recommendation given around March 1^st^ is followed by a final standardized test in April, after which (only upward) adjustments are possible, towards the final recommendation in early May. Schools are not obliged to adjust the recommendation. Before the pandemic, upward adjustments of school recommendations were disproportionately given to students of socioeconomically disadvantaged and migration backgrounds^38^. This finding is in keeping with a well-known hypothesis that standardized tests reduce inequalities^39^. The final standardized test was canceled in the first pandemic year, 2020, and the preliminary recommendations were not adjusted. Hence, the first outcome variable is the (final) recommendation given before the pandemic outbreak. Reports have described that, in 2020, the recommendations were lower than in previous years, especially for students of socioeconomically disadvantaged and migration backgrounds^38^. The interpretation of our first dependent variable is, then, the omitted opportunity to have one’s recommendation level adjusted due to the cancellation of the final school test, an opportunity that would, expectedly, have disproportionately benefited students of disadvantaged backgrounds. The track recommendations are (0) prevocational basic level (*vmbo-b*), (1) prevocational basic/cadre (*vmbo-b/k*), (2) prevocational cadre (*vmbo-k*), (3) prevocational cadre/theoretical (*vmbo-k/t*), (4) prevocational theoretical (*vmbo-t*), (5) prevocational theoretical/intermediate general (*vmbo-t/havo*), (6) intermediate general (*havo*), (7) intermediate general/pre-university (*havo/vwo*), and (8) pre-university (*vwo*).

The second variable is the enrolled school level at the start of secondary school (seventh grade). Because the Dutch system is rather complex at the first one or two years of secondary school, with many different options and track combinations, that do not neatly match the recommendation levels, the track enrolled in the first year is measured in eight categories. We ranked the categories based on the average primary school test scores in grades 4-5, which approaches linearity using the following coding: (0) prevocational basic (*vmbo-b*), (1) prevocational basic/cadre (*vmbo-b/k*), (2) prevocational cadre (*vmbo-k*), (3) comprehensive lower (*vmbo*/*havo* or *vmbo*/*havo*/*vwo*); (4) prevocational theoretical (*vmbo-t*), (5) intermediate general (*havo*), (6) comprehensive higher (*vmbo-t/havo/vwo* or *havo/vwo*), and (7) pre-university (*vwo*). We excluded students enrolled in “practical education” for whom the prevocational tracks are considered inadequate. Practical education focuses on students with IQ levels between 55 and 80, and with at least three years of delay in mathematics and literacy skills. We also excluded students who received a primary school recommendation for practical education or other forms of special education. In total, 1 percent of the students in the combined LVS/NCO data are omitted from the dataset for this reason.

The third dependent variable is the track in the third year of secondary school (i.e., ninth grade). At that stage, the three major tracks are (0) prevocational (*vmbo*), (1) intermediate (*havo*), and (2) pre-university track (*vwo*). Around four percent of ninth-grade students are still enrolled in a broad track including all three tracks, according to the registers. These are placed in the intermediate category. Distinguishing the seventh and ninth grade is relevant because schools vary a lot in the (single or mixed) tracks offered at the beginning of secondary school, while in the ninth grade, students are usually sorted into the three major tracks existing in the Dutch system. The pandemic effect is calculated for all three outcomes by subtracting the expected outcome from the actual outcome, following Eq. (3).

**Fig. 6:**
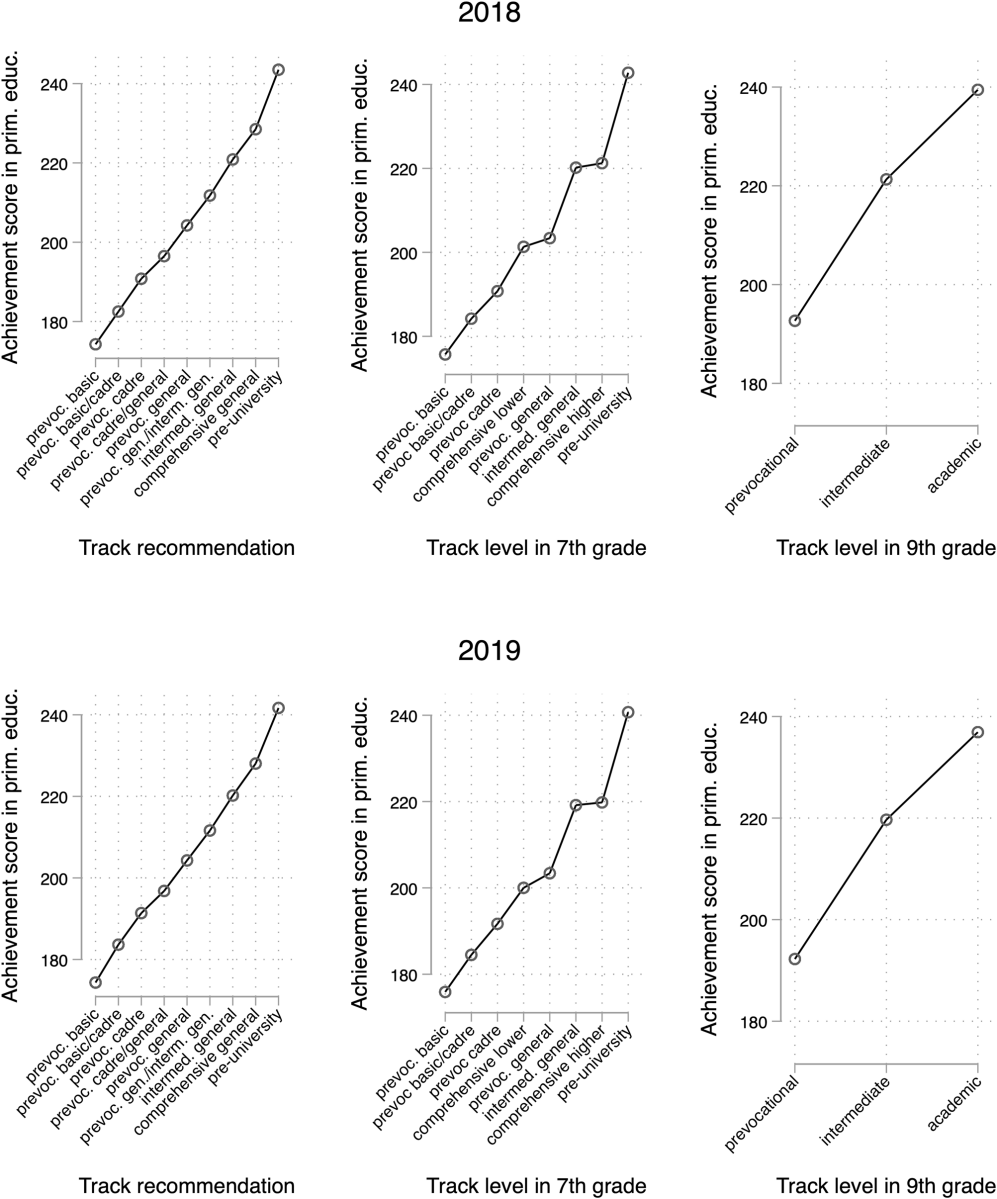
Three outcome variables, by primary school test scores in grades 4-5. This figure shows the ranked dependent variables in terms of track recommendation or enrollment rank based on student test scores in primary school, separately for the treatment (2019) and control cohort (2018).

The three outcomes are displayed based on the primary school achievement in Fig. 6. The track recommendation level follows a near-linear pattern based on primary school test scores. The seventh-grade track level also follows a monotonous distribution, with two deviations from a broad linear pattern. The prevocational theoretical track has a primary school achievement score similar to the comprehensive lower track. Also, intermediate general and comprehensive higher (bridging intermediate general and pre-university education) have somewhat similar primary school achievement scores. The track level in ninth grade also follows a rather linear pattern based on primary school test scores. Notably, the average scores of students in the pre-university track are somewhat lower in the ‘treatment cohort’ 2019 that went through the transition during the pandemic than in the previous cohort. In the discussion, we reflect on this finding.

### Other variables

Following our interest in socioeconomic gradients in the role of embeddedness, we use a combination of parents’ education and household income quintile (the highest income of either parent is taken in case of non-coresidence). Using education and income of the parents reflects the idea that these refer to different resources in the family of origin, as sociologists have long recognized. Parents’ education is measured by whether at least one of the parents has a degree in higher education. We categorize households in four distinct categories: both at least one parent with a college degree and a household income in any of the two highest quintiles; at least one parent with a college degree but household incomes in the middle quintile or less; no parent with a college degree and a household income at the middle quintile or higher; no parent with a college degree and a household income in the two poorest quintiles. With this categorization, we achieved a sufficient number of observations even when we rely on the smaller survey data that are merged with the register data.

Academic embeddedness is assessed with three latent factors consisting of Likert-type items ranging from 1-5: student *efficacy* (four items, including “I can even make the most difficult tasks if I give it my best” and “I am able to also learn difficult things at school”); student *motivation* (four items, including “I think school is interesting” and “When I wake up in the morning, I feel like going to school”); student *grit* (four items, including “I continue working even if things are not going so well” and “I give up if I lose track,” reverse coded).

Social embeddedness is assessed with parental involvement and parental network centrality. Parental involvement is measured using a five-item latent factor, including “My parent(s)/caregiver(s) ask me what I am learning at school,” “My parent(s)/caregiver(s) check if I have finished my school tasks (e.g., preparing a presentation).” To measure parental network centrality, we use sociometric data on parental contact, providing students with a roster with the names of all classmates. For each classmate, students were asked to report if their parent(s) occasionally talk to this peer’s parents. Based on the undirected classroom network, we calculate for each node (parent) its eigenvector centrality, which indicates the extent to which a parent is connected to many well-connected other parents (in network terminology this is seen as a measure of centrality, or influence). To avoid potential social disparities in self-reporting of network ties to affect the results, we do not take the directionality of ties into account. This implies that we consider all nominations, reciprocated or not, to signal parental contact. More information on the scale constructions is available in the PRIMS data documentation^32^.

## Supplementary information

Supplementary Information

## Data Availability

The analyses are based on calculations by the authors using non-public microdata from Statistics Netherlands. These Dutch administrative microdata can be accessed via the following link: https://www.cbs.nl/en-gb/onze-diensten/customised-services-microdata/microdata-conducting-your-own research. The microdata were analyzed via a secure internet connection (https://www.cbs.nl/en-gb/our-services/customised-services-microdata/microdata-conducting-your-own-research/rules-and-sanctioning-policy) after receiving authorization from Statistics Netherlands (CBS). For further details regarding CBS microdata access, please email: microdata@cbs.nl.

## Code availability

A replication package for this paper is available at https://osf.io/zf25j/. We can only publish statistical code, not the data (see above under Data Availability).
