# Supplementary material for: Inequality in pandemic effects on school track placement and the role of social and academic embeddedness
Source: NPJ Sci Learn. 2024 Nov 27;9:71. doi: 10.1038/s41539-024-00283-1 (PMC11603330; doi:10.1038/s41539-024-00283-1)
Supplement: Supplementary file 1 — Supplementary Information [file 41539_2024_283_MOESM1_ESM.pdf]

Inequality in pandemic effects on school track placement and the role of social and academic embeddedness

Online Supplement

Herman G. van de Werfhorst [a] *

Dieuwke Zwier [b]

Sara Geven [b]

Thijs Bol [b]

Carla Haelermans [c]

[a] European University Institute

[b] University of Amsterdam

[c] Maastricht University, Netherlands Initiative for Education Research (NRO), and NOLAI, Radboud University

* Corresponding author: Herman.vandeWerfhorst@eui.eu

Replication materials: https://osf.io/zf25j/

In this Online Supplement we provide additional relevant statistics supporting the analysis of the main paper. We focus on two topics: (1) a comparison of the mean values on key variables in the various subsamples, using t-tests (it should be noted that we use entropy balancing to match the distributions of the smaller PRIMS data to the LVS register data, as mentioned in the paper). (2) We show the size of the pandemic effect for the register data and the PRIMS subsample. (3) We present details of the measurement models underlying the structural equation models on the relationship between embeddedness variables and the size of the pandemic effects, and show fit statistics of the multigroup analysis by socioeconomic background.

1. Comparing distributions of key variables in subsamples

The requested overlap between the NCO, LVS and PRIMS data gives us a dataset of N=402 students. On the basis of t-tests of group differences on key variables, reported in Supplementary table 1, we perform the following tests:

(a) We test whether the LVS subsample differs from the population data from NCO, with regard to family background (parents' education, household income, migration status, and the dependent variables explained below). Panel A of Supplementary table 1 show that there are slightly more students with a parent with a college degree (about 1-2 percentage point difference between the LVS subsample and the population data for the two cohorts).

(b) We test whether the treatment and control cohorts of the combined LVS-NCO data are similar with regard to student background (parents' education, household income, migration status, and school performance). The results in Supplementary table 1 Panel B show that the gender distribution is almost identical. In the 2019 cohort there are slightly more parents with a college degree (a statistically significant difference of 0.7 percentage points). Moreover, (averaged) test scores in grades 4 and 5 are slightly lower in the treatment cohort than in the control cohort. In magnitude, these differences are very small though. The three outcome variables are significantly lower for the treatment cohort than for the control cohort, noting that these are raw differences.

(c) We test whether the total PRIMS sample is a representative sample of the LVS sample with regard to student background (parents' education, household income, track recommendation level, and the track levels in seventh and ninth grade). (Panel C in Supplementary table 1). It appears that the PRIMS sample is slightly skewed to more students with one or two parents with a college degree, and slightly higher school recommendations. The mean household income and the average secondary school track were not significantly different between the PRIMS and LVS data. The size of the estimated pandemic effect is more strongly negative for the PRIMS sample than for the joint LVS-NCO data.

(d) Lastly, we tested whether the analytical sample (i.e. the subsample of PRIMS that overlaps with the LVS sample) differs from the total PRIMS sample, on student background variables and the variables indicating academic and social embeddedness (Panel D). The percentage of students with parents' with a college degree is slightly higher in the analytical sample. Other variables, including the ones measuring academic and social embeddedness, do not significantly vary between the total PRIMS sample and the analytical sample.

Supplementary Table 1: T-tests of samples and populations

	Female	***Parents degree***	***Household income***	***Track recomm. level***	***Track level 7th grade (ranked)***	***Track level 9th grade (ranked)***					
						Panel A					
Mean of NCO 2018	0.500	0.459	72.488	4.960	4.714	0.920					
Mean LVS 2018 subsample	0.502	0.481	72.567	5.070	4.808	0.948					
t-value of difference	0.993	9.458	0.795	9.956	10.290	6.787					
p value	0.321	0.000	0.426	0.000	0.000	0.000					
Mean of NCO 2019	0.497	0.471	72.443	4.781	4.612	0.887					
Mean LVS 2019 subsample	0.498	0.488	72.325	4.825	4.640	0.893					
t-value of difference	0.184	7.730	-1.223	4.067	3.032	1.582					
p value	0.854	0.000	0.221	0.000	0.002	0.114					
						Panel B					
							Average reading score grades 4-5	***Average math score grades 4-5***			
Mean LVS 2018 (control) cohort	0.502	0.481	72.567	5.070	4.808	0.948	185.172	244.484			
Mean LVS 2019 (treatment) cohort	0.498	0.488	72.325	4.825	4.640	0.893	182.602	242.110			
t-value of difference	1.337	-2.346	1.744	15.804	13.005	9.470	15.692	13.543			
p value	0.181	0.019	0.081	0.000	0.000	0.000	0.000	0.000			
						Panel C					
							Pandemic effect on track recomm. level	***Pandemic effect on track 7th grade***	***Pandemic effect on track 9th grade***		
Mean LVS 2019 cohort	0.498	0.488	72.348	4.825	4.640	0.893	-0.068	-0.039	0.011		
Mean PRIMS subsample	0.524	0.523	72.744	5.204	4.773	0.970	-0.124	-0.227	-0.050		
t-value of difference	1.852	2.412	0.668	3.155	1.335	1.718	-0.952	-3.061	-2.081		
p value	0.064	0.016	0.505	0.002	0.183	0.087	0.342	0.002	0.038		
						Panel D					
							Efficacy	***Motivation***	***Grit***	***Parental involvement***	***Centrality in parental network***
Mean PRIMS total sample	0.524	0.523	72.744				0.000	0.000	0.000	0.000	0.000
Mean PRIMS analytical sample	0.545	0.587	70.715				0.034	-0.043	0.014	0.059	0.054
t-value of difference	0.821	2.617	-1.852				0.660	-0.786	0.269	1.253	1.018
p value	0.412	0.009	0.065				0.510	0.432	0.788	0.211	0.309

Supplementary Figure 1: Estimated pandemic effects for the register data and the PRIMS sample. This figure shows the estimated pandemic effects on three outcomes for the register data and for the subsample of the PRIMS survey, showing largely similar, but slightly more negative pandemic effects for the subsample.

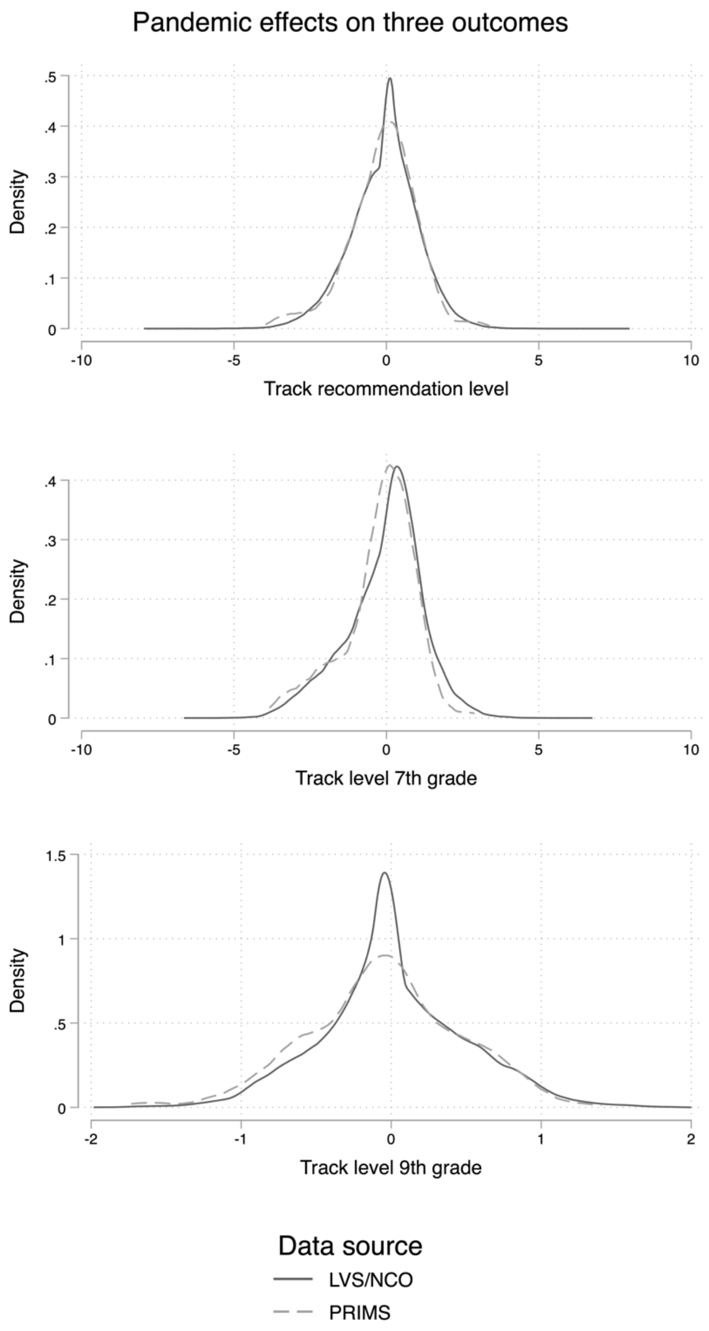

2. Pandemic effect by data source

Supplementary Figure 1 shows the pandemic effects for the PRIMS subsample and the NCO/LVS register data. These effects are measured in units on the educational ladders described in the Methods section. As the figure shows, the distributions of the pandemic effects are very similar between the NCO/LVS sample and the much smaller PRIMS data with regard to the track recommendation level. The pandemic effect on track level in seventh and ninth grade is slightly more negative in the PRIMS-data than in the NCO/LVS sample.

3. Structural equation models: measurement models

We conducted confirmatory factor analyses (CFA) for all scales with multiple items (i.e., student efficacy, motivation and grit, and parental involvement) using the structural equation package <sem> in Stata 16. Moreover, we tested these factors for measurement invariance across student SES. To be able to compare students' mean scores on these factors across tracks, (partial) scalar invariance is required.[1] To test for measurement invariance, we follow the steps described for Stata.[2] That is, we first tests for equality of the measurement coefficients and, subsequently, test for equality of item intercepts.

To assess the model fit, we use the comparative fit index (CFI), the Tucker-Lewis Index (TLI), and the root mean square error of approximation (RMSEA). We use cut-off values of >.95 and >.90 for the CFI and TLI and a cut-off of <.05 for the RMSEA.[3] Based on theoretical considerations and Stata's modification indices, we examine how measurement models can be improved in case these cut-off values are not reached. When testing for measurement invariance, we rely on a Wald test of the $\Delta\ \chi2$. Measurement invariance is rejected when the $\Delta\ \chi2$ between the restricted and less restricted model is statistically significant.

For student efficacy and motivation, we find a good model fit for a 4-item factor (efficacy: RMSEA<0.000, CFI=1.00, TLI=1.00; motivation: RMSEA=0.000, CFI=1.000, TLI=1.013) and support for scalar invariance across the different SES groups (see Supplementary table 2).

For student grit, a satisfactory model fit is not obtained for a 4-item factor (RMSEA=0.092, CFI=0.972, TLI=0.916). Modification indices suggest to add a covariance between the error term of the item "I continue working even if things are not going so well" and "I dare to get

started even if things might go wrong". As these items seem theoretically related, we add this covariance, after which we find a satisfactory model fit for this measurement model (RMSEA=0.036, CFI=0.998, TLI=0.987). Moreover the model fit of a model in which both measurement coefficients and intercepts are constrained is not satisfactory for grit. However, Δ χ2 tests of the comparison of this model to a model in which only the coefficients are constrained to be equal across SES groups is not statistically significant (see Supplementary Table 2). This indicates that we cannot reject the null hypothesis that intercepts are also equal across the groups.

Supplementary Table 2: Fit statistics of measurement models

		Model without constraints	Model with equal measurement coefficients across SES groups	Model with equal measurement coefficients and intercepts across SES groups
Efficacy	χ2	χ2(8)=10.133, p=0.256)	χ2(20)=22.763, p=0.301)	χ2(32)=33.812, p=0.380)
	Δ χ2 (v.s. less restrictive model)		Δ χ2 (12)=12.741, p=0.3381)	Δ χ2 (12)=11.201, p=0.5117)
	CFI	0.996	0.995	0.996
	TLI	0.987	0.994	0.997
	RMSEA	0.052	0.037	0.024
Motivation	χ2	χ2(8)=5.374, p=0.717)	χ2(20)=8.901, p=0.984)	χ2(32)=20.960, p=0.933)
	Δ χ2 (v.s. less restrictive model)		Δ χ2 (12)=3.508, p=0.9908)	Δ χ2 (12)=12.171, p=0.4321)
	CFI	1.000	1.000	1.000
	TLI	1.021	1.035	1.022
	RMSEA	0.000	0.000	0.000
Grit	χ2	χ2(4)=3.030, p=0.553)	Δ χ2 (16)=18.216, p=0.311)	Δ χ2 (28)=35.815, p=0.147)
	Δ χ2 (v.s. less restrictive model)		Δ χ2 (12)=14.306, p=0.2816)	Δ χ2 (12)=17.889, p=0.1191)
	CFI	1.000	0.991	0.968
	TLI	1.024	0.986	0.972
	RMSEA	0.000	0.037	0.053
Parental involvement	χ2	χ2(12)=22.544, p=0.032)	χ2(27)=47.024, p=0.010)	Δ χ2 (42)=72.107, p=0.003)
	Δ χ2 (v.s. less restrictive model)		Δ χ2 (15)=24.152, p=0.0626)	Δ χ2 (15)=25.682, p=0.0415)
	CFI	0.968	0.939	0.908
	TLI	0.892	0.909	0.912
	RMSEA	0.095	0.087	0.086

For parental involvement the initial model fit of a 5-item factor is non-satisfactory (RMSEA=0.113, CFI=0.921, TLI=0.843). Modification indices suggest to add covariances between the items "Parent(s)/caregiver(s) ask me about what I am learning in school" and "Parent(s)/caregiver(s) talk with me about what I did at school" as well as between "Parent(s)/caregiver(s) talk with me about my secondary school choice" and "parent(s)/caregiver(s) check if I have finished my school tasks". After adding these, the model fit is good (RMSEA=0.000, CFI=1.000, TLI=1.011). For parental involvement we do not find support for full measurement invariance. A model in which all measurement intercepts and coefficients are constrained fits worse than a model in which only the coefficients are constrained. For two of the five items we find differences between the intercepts across the groups (i.e., parents talk about secondary school choice and parents make sure I take the time to carry out school tasks). Hence, partial scalar invariance is obtained (i.e., at least two loadings and intercepts are constrained equal across the groups) and latent factor means can be compared.

Supplementary Table 3 shows the fit statistics for the multigroup analysis compared to the single-group analysis. According to the chi-square likelihood ratio test and the Akaike Information Criterion, the model with socioeconomic group-specific parameters of the embeddedness variables fits the data better than the general model. According to the BIC criterion, there is sometimes a slight preference for the general model. We conclude that it is valuable to explore differences between socioeconomic groups in the embeddedness correlates of pandemic effects.

Supplementary Table 4 shows the parameter estimates as they are displayed in Figures 3 and 4 of the main paper.

Supplementary Table 3: Fit statistics of multigroup models versus general models (measurement model and structural model)

	General				SES-specific						
	df	LL	AIC	BIC	df	LL	AIC	BIC	-2LL Chi sq test	df	sig
					Track recommendation						
Efficacy	19	-3318.95	6675.89	***6751.82***	61	***-3215.64***	***6553.28***	6797.06	206.614	42	***
Motivation	19	-3441.57	6921.14	***6997.08***	61	***-3347.07***	***6816.14***	7059.93	189.002	42	***
Grit	18	-3436.79	6909.58	***6981.51***	65	***-3301.99***	***6733.98***	6993.75	269.598	47	***
Parental involvement	24	-3634.55	7317.11	***7413.02***	71	***-3520.68***	***7183.35***	7467.10	227.752	47	***
Network centrality	9	-2013.76	4045.52	4081.49	27	***-1930.95***	***3915.91***	***4023.81***	165.614	18	***
					Secondary school track seventh grade						
Efficacy	19	-3207.59	6453.19	***6529.12***	61	***-3098.38***	***6318.75***	6562.53	218.438	42	***
Motivation	19	-3329.15	6696.30	***6772.24***	61	***-3228.16***	***6578.31***	6822.09	201.992	42	***
Grit	18	-3322.23	6680.47	***6752.40***	65	***-3185.70***	***6501.40***	6761.16	273.072	47	***
Parental involvement	24	-3521.63	7091.26	***7187.18***	67	***-3403.06***	***6940.11***	7207.87	237.15	43	***
Network centrality	9	-1895.27	3808.53	3844.50	27	***-1813.66***	***3681.33***	***3789.23***	163.204	18	***
					Secondary school track ninth grade						
Efficacy	19	-2450.99	4939.98	***5015.91***	61	***-2337.44***	***4796.88***	5040.66	227.102	42	***
Motivation	19	-2573.70	5185.41	***5261.34***	61	***-2469.67***	***5061.34***	5305.12	208.068	42	***
Grit	18	-2565.20	5166.40	5238.34	65	***-2423.16***	***4976.32***	***5236.09***	284.084	47	***
Parental involvement	24	-2765.04	5578.08	***5673.99***	71	***-2643.04***	***5428.08***	5711.83	244.002	47	***
Network centrality	9	-1143.03	2304.07	2340.03	27	***-1050.25***	***2154.50***	***2262.41***	185.564	18	***

*** p<0.001

In ***bold-italic*** : preferred model according to this statistic

Supplementary Table 4: Parameter estimates of embeddedness effects as displayed in Figures 3 and 4

	Averaged over all students			Lower income and no parent with a college degree			Medium/high income, no parent with a college degree			Lower income, at least one parent with a college degree			Higher income, at least one parent with a college degree		
Variable	b	CI left	CI right	b	CI left	CI right	b	CI left	CI right	b	CI left	CI right	b	CI left	CI right
							Track recommendation level								
Efficacy	0.146	0.064	0.229	0.192	0.039	0.345	0.133	0.018	0.249	0.158	0.028	0.288	0.166	0.027	0.305
Motivation	0.121	0.056	0.187	0.181	0.004	0.358	0.100	-0.027	0.227	0.115	0.032	0.199	0.123	0.007	0.239
Grit	-0.043	-0.110	0.024	-0.002	-0.120	0.115	0.035	-0.132	0.203	-0.107	-0.234	0.020	-0.053	-0.184	0.078
Parental involvement	0.075	0.009	0.142	0.072	-0.037	0.182	0.053	-0.089	0.195	0.148	0.014	0.282	0.051	-0.069	0.172
Network centrality	0.006	-0.061	0.072	-0.003	-0.100	0.094	-0.012	-0.173	0.149	0.036	-0.070	0.143	0.050	-0.053	0.153
							Track level seventh grade								
Efficacy	0.166	0.066	0.265	0.223	0.031	0.415	0.134	-0.046	0.315	0.172	0.000	0.344	0.248	0.082	0.413
Motivation	0.123	0.036	0.211	0.022	-0.188	0.232	0.208	0.077	0.340	0.140	0.025	0.254	0.193	0.055	0.332
Grit	-0.039	-0.155	0.076	0.023	-0.148	0.194	0.030	-0.251	0.312	-0.109	-0.266	0.048	-0.104	-0.304	0.097
Parental involvement	0.014	-0.073	0.101	-0.159	-0.335	0.017	0.104	-0.088	0.295	0.080	-0.051	0.211	0.129	-0.054	0.313
Network centrality	0.027	-0.050	0.104	0.016	-0.128	0.160	0.001	-0.148	0.149	0.080	-0.058	0.217	-0.009	-0.153	0.135
							Track level ninth grade								
Efficacy	0.123	0.019	0.228	0.218	0.038	0.398	0.249	0.060	0.438	0.101	-0.092	0.295	0.157	0.012	0.302
Motivation	0.065	-0.010	0.141	0.073	-0.072	0.218	0.111	-0.002	0.223	0.086	-0.045	0.216	0.149	0.067	0.230
Grit	0.047	-0.051	0.144	0.117	-0.084	0.318	0.067	-0.222	0.356	-0.027	-0.217	0.164	-0.038	-0.168	0.093
Parental involvement	0.027	-0.056	0.109	0.122	-0.030	0.273	-0.037	-0.252	0.178	0.035	-0.099	0.169	-0.042	-0.224	0.140
Network centrality	-0.018	-0.089	0.053	-0.081	-0.234	0.073	0.045	-0.132	0.223	0.016	-0.132	0.165	-0.083	-0.238	0.073

Estimated regression coefficients and confidence intervals of embeddedness variables, holding constant for the counterfactual predicted outcome in the absence of a pandemic

Supplementary References

1. van de Schoot, R., Lugtig, P. & Hox, J. A checklist for testing measurement invariance. *European Journal of Developmental Psychology* **9**, 486–492 (2012).
2. MacDonald, K. Group comparisons in structural equation models: Testing measurement invariance. *The Stata Blog* http://blog.stata.com/2016/08/23/group-comparisons-in-structural-equation-models-testingmeasurement-invariance/ (2016).
3. Hu, L. & Bentler, P. M. Cutoff criteria for fit indexes in covariance structure analysis: Conventional criteria versus new alternatives. *Structural Equation Modeling: A Multidisciplinary Journal* **6**, 1–55 (1999).
